# A bibliometric analysis of the application of physical therapy in knee osteoarthritis from 2013 to 2022

**DOI:** 10.3389/fmed.2024.1418433

**Published:** 2024-09-03

**Authors:** Chenglan Huang, Yutong Hou, Yunxiao Yang, Jiaqi Liu, Ya Li, Dezhi Lu, Sha Chen, Jinwu Wang

**Affiliations:** ^1^School of Rehabilitation, Shandong Second Medical University, Weifang, China; ^2^School of Medicine, Shanghai University, Shanghai, China; ^3^Department of Acupuncture and Rehabilitation, The 2nd Affiliated Hospital of Guizhou University of Traditional Chinese Medicine, Guiyang, China; ^4^Shanghai Key Laboratory of Orthopaedic Implants, Department of Orthopaedic Surgery, Shanghai Ninth People’s Hospital, Shanghai Jiao Tong University School of Medicine, Shanghai, China

**Keywords:** physical therapy, knee osteoarthritis, CiteSpace, VOSviewer, visual analysis

## Abstract

**Background:**

Knee osteoarthritis (KOA) is one of the most common chronic joint diseases. Physical therapy, a non-invasive approach, is extensively used in its treatment. Although bibliometrics is a reliable method to evaluate the significance and impact of research fields, systematic bibliometric analyses in this area are lacking. This study aims to perform a bibliometric analysis covering 2013 to 2022, to highlight the current state, key focuses, and trends in physical therapy for KOA.

**Methods:**

This study utilizes the Web of Science Core Collection to gather relevant literature on physical therapy and KOA from 2013 to 2022. CiteSpace and VOSviewer software facilitated the visual analysis of the annual publications, geographic and institutional distributions, journals, authors, references, and keywords in this field.

**Results:**

The study analyzed 1,357 articles, showing an overall increase in publications over time from 71 countries and 2,302 institutions. The United States and Australia emerged as leaders in this field. The analysis identified 6,046 authors, with Kim L. Bennell as the most prolific and Bellamy N. receiving the most citations. BMC Musculoskeletal Disorders published the most articles, while Osteoarthritis and Cartilage received the most citations. High-impact articles were authored notably by McAlindon TE, Bannuru RR, Fernandes L, and Bennell KL. Keyword analysis highlighted a strong focus on patient self-management, exercise therapy, physical factor therapy, and remote rehabilitation.

**Conclusion:**

The bibliometric analysis confirms significant interest and ongoing research in physical therapy for KOA treatment from 2013 to 2022, indicating a growing field. Journals and authors in this area show influential and collaborative dynamics. Future research should focus on enhancing international and institutional collaboration and explore emerging trends like internet-guided treatments.

## 1 Introduction

Osteoarthritis is one of the most common joint diseases, particularly affecting the knees, hands, and hips (1, 2). Knee osteoarthritis (KOA) is a significant orthopedic condition primarily caused by mechanical cartilage wear and joint dysfunction, leading to symptoms such as pain, stiffness, numbness, and reduced mobility (3–5). Currently, about 6.541 million individuals are affected by KOA, which accounts for 8.1% of the population. Various factors influence its prevalence, such as gender, age, weight, socioeconomic status, education level, and comorbidities, including cardiovascular and gastrointestinal diseases (6–10). Moreover, factors like increased life expectancy, an aging demographic, and rising obesity rates exacerbate the clinical and economic impacts of KOA, posing major public health challenges (11–13). Consequently, the development of effective treatments is critical and includes pharmacological methods, surgical options, and non-pharmacological strategies (14). Although commonly used, medications such as opioids and NSAIDs are associated with significant risks, including gastrointestinal, cardiovascular, and renal complications (15–17). Surgical interventions are usually considered a last resort due to the associated risks of infection and thrombosis (18). Current guidelines prioritize non-pharmacological interventions such as exercise therapy, patient education, and weight management as primary treatments (19–21). As a component of non-pharmacological treatment, physical therapy includes exercise therapy and physical factor therapy. These non-invasive treatments for KOA aim to relieve pain, improve function, and slow disease progression, significantly enhancing patients’ quality of life (22–24).

Bibliometrics, founded by American bibliographer Alan Pritchard in 1969, combines mathematics, statistics, and documentation to analyze literature both qualitatively and quantitatively. This field facilitates a deep understanding of the knowledge structure within specific research areas and aids in identifying developmental trends (25, 26). Prominent tools for bibliometric analysis include CiteSpace and VOSviewer. CiteSpace was developed by Professor Chaomei Chen at Drexel University, USA, and VOSviewer was created by Nees Jan van Eck and Ludo Waltman in 2010. These Java-based applications specialize in bibliometric, co-occurrence, and cluster analyses, effectively visualizing research trends and critical areas across various disciplines over designated timeframes. They analyze data from different countries, institutions, and authors, and map keywords and co-citation networks that include journals, authors, and publications (27–30).

Bibliometric analysis is crucial for advancing research in the medical field. It reveals research trends and hotspots, measures the impact of research results, identifies academic networks and collaborations, detects research gaps, and provides a scientific basis for policy formulation. Currently, bibliometric studies on KOA include topics such as *Platelet-rich plasma in osteoarthritis of the knee* and *Intra-articular injection therapy for osteoarthritis of the knee* (31, 32). However, there are no bibliometric studies specifically examining the use of physical therapy in KOA. Therefore, this study aims to systematically analyze recent studies on the application of physical therapy in KOA using CiteSpace and VOSviewer, to explore the current status, hotspots, and development trends, and to provide guidance and direction for future research.

## 2 Materials and methods

### 2.1 Literature sources

The Web of Science Core Collection is widely recommended as the preferred database for bibliometric analyses due to its rigorous evaluation process and high-impact, trustworthy information (33). Numerous successful bibliometric studies have been conducted using data from the Web of Science Core Collection (34, 35). In the Web of Science (WOS) database, TS stands for “Topic” search, a Boolean logic-based method that uses subject terms to quickly and easily retrieve a large amount of topic-related information.

In this study, data were retrieved from the Web of Science Core Collection, which includes the Science Citation Index Expanded (SCI-EXPANDED), Social Sciences Citation Index (SSCI), Conference Proceedings Citation Index-Science (CPCI-S), and Index Chemicus (IC). Searches were conducted using “physical therapy” and “knee osteoarthritis” as the main subject terms. The search strategy incorporated multiple related keywords and synonyms to ensure comprehensiveness (refer to Supplementary Table 1). The search period spanned from January 1, 2013, to December 31, 2022, with the search language set to “English” and the document type restricted to “article.” A total of 1,852 articles were retrieved, and 1,357 articles were included after deduplication. The specific search strategy and results can be found in Supplementary Figure 1.

### 2.2 Data processing

The retrieved literature was exported in plain text format, comprising full records and cited references, and stored in a designated folder named “download_.txt.” Subsequently, we imported this data into CiteSpace 6.2.R4. Duplicate literature was diligently removed, and we then generated scientific knowledge maps for in-depth analysis using both CiteSpace and VOSviewer.

### 2.3 Parameter settings

In CiteSpace, we configured the time span to cover the years 2013 to 2022, with the default time zone segmentation set at “1” year. The topic term was left in its default setting of full selection. Co-occurrence analyses were performed on journals, countries, institutions, authors, and keywords. Additionally, co-citation analyses were conducted for journals, authors, and literature. The threshold parameter was established at Top N = 50, and for pruning methods, including institutional cooperation analysis, network analysis, journal co-citation, and author cooperation analysis, we selected the Pathfinder method. We visualized keyword clustering using VOSviewer. Furthermore, we analyzed the co-occurrence of countries employing CiteSpace, VOSviewer, and Scimago Graphica. Descriptive analysis of bibliometric indicators, encompassing publication volume, countries of origin, affiliated institutions, journals of publication, contributing authors, cited references, and keywords, was carried out using Microsoft Excel 2016.

### 2.4 Main observation indicators

We conducted a comprehensive discussion and analysis of publication volume, countries of origin, affiliated institutions, journals of publication, contributing authors, cited references, and keywords. This analysis was informed by the relevant scientific knowledge maps generated using CiteSpace, VOSviewer, and Scimago Graphica, and it was complemented by data extracted from the Web of Science database.

## 3 Results

### 3.1 Trends in publication volume

From 2013 to 2022, a total of 1,357 papers were published in this field. Specifically, from 2013 to 2014, the number of publications decreased from 94 to 79. However, 2015 to 2016 witnessed an increase in publications, but in 2017, there was another decline, with 30 fewer publications than in 2014. Beginning in 2017 and continuing until 2020, there was a consistent upward trend in the number of publications, reaching its peak in 2020. Despite a slight decrease in publications in 2021 and 2022 compared to 2020, the number of publications in 2021 exceeded that of 2019, and the count for 2022 was close to that of 2020. Additionally, an analysis using the citation report feature in the Web of Science database reveals a total citation count of 23,457. This count increased significantly, from 113 citations in 2012 to a peak of 5,381 citations in 2022. The average citation count per paper is 17.1, and the H-index is 61.

### 3.2 Distribution of countries/regions and institutions

A total of 1,357 relevant papers were published by 2,302 institutions from 71 different countries. Supplementary Table 2 reveals that the highest number of publications originated from the USA (412, 30.36%), significantly surpassing Australia (151, 11.13%) and China (120, 8.84%). The institution with the highest publication count is the University of Melbourne (72, 5.31%). Remarkably, among the top-10-ranked research institutions, 70% belong to the USA. Furthermore, certain countries and institutions demonstrate relatively high centrality, including the USA (0.56), Australia (0.16), England (0.18), the Netherlands (0.13), the University of Melbourne (0.13), and Harvard University (0.14). In Supplementary Figures 3, 4, each circle represents a country or institution, and the circle’s size indicates their publication output. The colorful lines connecting the circles signify the strength of collaboration between countries or institutions, with thicker lines indicating closer collaboration. However, certain countries and research institutions appear scattered, lacking stable and close collaborative and communication relationships.

### 3.3 Journals and co-cited journals

We conducted a visual analysis of published journals using VOSviewer software and discovered a total of 362 academic journals that have published articles related to this field. Among them, *BMC Musculoskeletal Disorders* (84, 6.53%) had the highest output, followed by *Arthritis Care & Research* (40, 3.11%). Among the top 10 academic journals, *Osteoarthritis and Cartilage* boasted the highest Impact Factor (IF) at IF = 7.0. Furthermore, as shown in Supplementary Table 3, 60% of the top 10 journals by publication volume are in Q1, 30% are in Q2, and 10% are in Q3.Regarding the citation counts of journals, as displayed in Supplementary Table 3, six journals had citation counts exceeding 500, with *Osteoarthritis and Cartilage* (911) being the most frequently cited, followed by *Arthritis Care & Research* (857).Among the co-cited journals, *Annals of the Rheumatic Diseases* had the highest IF. According to the 2022 Journal Citation Reports (JCR), except for *Journal of Rheumatology* and *BMC Musculoskeletal Disorders*, nearly all the top 10 co-cited journals belonged to Q1.

In the dual-graph representation of journals, the left side depicts citing journals, and the right side represents cited journals. The relationships between them are depicted by the colored lines in the graph. The horizontal axis of the ellipses corresponds to the number of authors, while the vertical axis represents the number of journals. As shown in Supplementary Figure 5, the green and pink citation paths indicate that papers published in Sports/Rehabilitation/Sport and Health/Nursing/Medicine journals are often cited by journals in Medicine/Medical/Clinical and Neurology/Sports/Ophthalmology.

### 3.4 Authors and co-cited authors

A total of 6,046 authors have contributed to research on physical therapy applications in knee osteoarthritis. As shown in Supplementary Table 4, among the top 10 authors by publication volume, Kim L. Bennell (40 articles, 2.95%) has the highest publication count, with Kim L. Bennell (0.07) and Ewa M. Roos (0.05) displaying relatively high centrality. In Supplementary Figure 6, each circle represents a corresponding author, and the colored lines connecting them illustrate connections among authors. The diverse colors forming interconnected networks signify different author collaboration clusters, such as Hinman, Rana S.; Lawford, Belinda J.; Forbes, Andrew; Harris, Anthony; Kasza, Jessica; Abbott, J. Haxby; Allen, Kelli D.; Coffman, Cynthia J.; Oddone, Eugene Z.; and Huffman, Kim M. Co-cited authors refer to two or more authors simultaneously cited by one or more research papers, indicating a shared co-citation relationship. The most frequently co-cited author is Bellamy N (374), followed by Anonymous (287).

### 3.5 Co-cited references and references burst

Co-citation refers to the relationship between two or more articles that are simultaneously cited by one or more other articles. Supplementary Table 5 presents the top 10 most frequently cited references, consisting of eight guidelines and two reviews. The most highly cited reference is the article by McAlindon TE et al. (20), published in 2014. Based on references with the strongest citation bursts, Supplementary Figure 7 illustrates that co-cited references originated in 2013. The highest burst strength is attributed to Bannuru RR (strength = 33.82), and the longest burst duration belongs to the articles authored by Fernandes L and Bennell KL.

### 3.6 Keyword co-occurrence, clusters, and timeline view

Supplementary Table 6 reveals that this study includes 18 keywords with a frequency of occurrence exceeding 100 times, Among them, keywords appearing more than 200 times include “knee osteoarthritis” (434), “hip” (354), “management” (290), “osteoarthritis” (276), “pain” (274), “physical therapy” (236), and “therapy” (205), Keywords with frequencies exceeding 130 times but less than 200 comprise “exercise” (176), “older adults” (146), and “quality of life” (131).

In this study, we used VOSviewer software to cluster the keywords, resulting in Supplementary Figure 8. In this visualization, distinct colors represent different clusters, each composed of colored circles representing keywords. The size of each circle reflects the frequency of that keyword’s occurrence in the articles, while the proximity of circles indicates the degree of association between keywords. Observing Supplementary Figure 8, it becomes apparent that the blue cluster primarily consists of knee osteoarthritis, physical therapy, and exercise therapy. The green cluster mainly includes older adults, physical function, and muscle strength. The yellow cluster mainly contains osteoarthritis, rehabilitation, and physical therapy, while the red cluster features pain, knee, and hip.

Supplementary Figure 9 illustrates the keyword timeline view generated by CiteSpace software. From 2012 to 2017, the key terms revolved around weight loss, efficacy, low-level laser, manual therapy, electrical nerve stimulation, spa therapy, and balneotherapy. In the period from 2017 to 2022, the main keywords shifted to muscle strength, mud pack therapy, aerobic exercise, home, neuromuscular exercise, patient satisfaction, ultrasound, home telerehabilitation, and decision making.

## 4 Discussion

### 4.1 General information

The annual publication trends can offer insights into the pace and progress of our study. As shown in Supplementary Figure 2, the number of annual publications experienced fluctuations between 2013 and 2022, but it indicated an overall upward trend. The period from 2013 to 2015 saw a lower publication output, indicating the early stage of research on the application of physical therapy for KOA. From 2015 to 2020, there was a noticeable fluctuation in publication numbers, indicating consistent growth. Notably, in 2020, there was a peak in publications, reaching the highest count in nearly 10 years, emphasizing increased attention to this research field during that period. Although there was a minor decrease in publication output from 2020 to 2022, it was relatively small, suggesting that interest persisted in 2021 and 2022, even though it did not reach the levels seen in 2020.

Supplementary Table 2 reveals that the USA has the highest number of publications (412, 30.36%), establishing it as the foremost country in this research domain. Centrality is a metric employed to gauge the significance of nodes within a network structure. It ranges from 0 to 1, with higher values denoting greater influence. When centrality surpasses 0.1, it signifies a pivotal node. Among the top 10 research countries, the USA (0.56) boasts the highest centrality score, signifying its pivotal role in the global collaborative network among nations. Seven out of the top 10 research institutions are American, with Harvard University exerting the most significant influence. Supplementary Figures 3, 4 reveal that only a handful of countries and institutions are dispersed, such as Turkey and Duke University, which fail to establish a network. This can impede research progress in this field. Consequently, it is advisable for all countries and their research institutes to actively engage in collaborative exchanges and advance research on the application of physiotherapy for KOA.

*BMC Musculoskeletal Disorders* had the highest number of publications (84, 6.53%), followed by *Arthritis Care & Research* (40, 3.11%). This underscores the current significance and future research trends in KOA and its management. Analyzing the distribution of literature sources aids in identifying core journals within this research area. *Osteoarthritis and Cartilage* (911), being the most highly cited journal, is recognized as a central journal in this field. Furthermore, a majority of the journals and cited sources were in the Q1 category, demonstrating that the published papers and citations originated from high-impact journals. This underscores the significant value of research on physiotherapy applied to KOA within the academic field.

In the analysis of authors and co-cited authors, Kim L. Bennell (40, 2.95%) from the University of Melbourne made the most significant contribution and had the highest impact. In 2022, Kim L. Bennell demonstrated improvement in pain and function among KOA patients through the provision of exercise and dietary programs via telemedicine (36). Furthermore, Kim L. Bennell offers medical care to individuals unable to access in-person healthcare facilities and has developed a complimentary PEAK online course. This course educates physiotherapists on implementing evidence-based exercise management programs for individuals with KOA (37). Bellamy N is the most frequently cited author (374 citations), while McAlindon TE possesses relatively high centrality (0.07), indicating their considerable influence in this research area.

### 4.2 The hotspots and frontiers

The top 10 cited references include eight guidelines. These guidelines are founded on consensus judgments of clinical experts from diverse disciplines, grounded in available evidence, and prioritize patient-centered care. They offer treatment guidance tailored to KOA patients, considering their unique needs and preferences. The analysis of emerging references signifies the emergence of potential research questions within a specific topic. In 2013, the cited references predominantly emphasized interventions for KOA patients, focusing on self-management, pharmacological treatments, and exercise therapies. These exercise therapies primarily comprised aerobic exercise, plyometrics, joint mobility, proprioception, and balance training. During this period, physical factors for KOA treatment received less attention within this field (38–41). In recent years, there has been a progressive shift towards focusing on patients’ mental health, orthotics, telerehabilitation, and physical factor therapy, in addition to the initial emphasis (20, 21, 42–44). Bannuru RR et al.’s study (20) had the highest citation burst intensity (33.82) and ranked first among the top 30 references, highlighting its milestone significance in 2021. The study supplemented the original guidelines with detailed recommendations for oral NSAIDs. It introduced tai chi and yoga as novel exercise therapies for KOA treatment and provided comprehensive insights into treatments with limited evidence, such as hydrotherapy, orthotics, and assistive devices. Fernandes L et al.’s work (45) led to the most enduring literature impact, advocating a comprehensive approach that integrates patient education, weight loss, and exercise therapy, facilitating the incorporation of exercise programs into daily life with progressively increasing intensity and duration. The study also specified the frequency of follow-up visits and treatment modalities. Bennell KL et al. (46) used physiotherapists to instruct patients in home rehabilitation via Internet videoconferencing. This indicates that integrating Internet-assisted rehabilitation exercises can reduce the burden of rehabilitation care while effectively improving patient function.

Keyword co-occurrence analysis reveals research hotspots, keyword clustering delineates the knowledge structure, and a timeline view of keywords illustrates changes over time, reflecting the evolution of research trends. In this study, clustering was conducted using keyword co-occurrence data, and a timeline view was generated based on the clustering results to identify research hotspots, trends, and developmental frontiers in the field of physiotherapy applied to KOA. Between 2012 and 2017, research primarily centered on evaluating the effectiveness of patient self-management, exercise therapy, and physiological factor therapy. Subsequently, from 2017 to 2022, research built upon prior work to enhance clinical treatment decision-making, with a focus on patient treatment satisfaction and the integration of tele-rehabilitation. While patient self-management is recommended as a core treatment for KOA by the Osteoarthritis Research Society International, research findings regarding its effectiveness are partly contentious. Some studies suggest that self-management improves pain, stiffness, knee function, self-efficacy, mental health, and quality of life in KOA patients (19, 47, 48). Conversely, other studies propose that self-management does not significantly enhance pain and joint function, possibly due to variations in social construction theories, research methodologies, and interventions within the sample (49, 50). Exercise therapy encompasses diverse therapeutic modalities, including individual, group, and family approaches. It is essential to devise personalized exercise treatment plans considering injury severity, patient preferences, complications, and adherence. Currently, international guidelines predominantly endorse aerobic exercise and lower limb muscle training, yet there is no standardized criterion for exercise dosage encompassing frequency, intensity, and duration. Conducting additional clinical trials is imperative to establish optimal exercise content and duration. Furthermore, assessing the long-term effectiveness of exercise therapy for KOA should be a focus in future research (51, 52). Physical factor therapy serves as a complementary approach for managing KOA, with the potential to enhance therapeutic outcomes and alleviate associated symptoms. However, the Osteoarthritis Research Society International and the American College of Rheumatology express reservations concerning the overall quality of the supporting evidence. Notably, transcutaneous electrical stimulation, a practice strongly discouraged by the Osteoarthritis Research Society International for KOA patients, illustrates this skepticism. Moreover, pertinent research indicates that exercise therapy may yield more enduring benefits for KOA patients compared to physical factor therapy. Numerous studies have concluded that exercise therapy surpasses physical factor therapy in delivering lasting relief to KOA patients. Consequently, a compelling imperative exists for conducting higher-quality clinical trials to evaluate the effectiveness of physical factor therapy for KOA (19, 20, 53). Against the backdrop of the widespread integration of the Internet into the medical domain, the approach to treating KOA has progressively shifted from traditional face-to-face methods to tele-rehabilitation. This transformative shift enables remote services such as online follow-up, health education, exercise regimens, and periodic evaluations for KOA patients. These interventions effectively ameliorate KOA-related symptoms and contribute to heightened patient satisfaction. Nevertheless, the approaches to Internet-based tele-rehabilitation have exhibited variances across studies, resulting in disparities in its efficacy. Consequently, forthcoming research endeavors should prioritize delivering higher-quality clinical studies to systematically address this variability (54–56).

## 5 Limitations

This study is subject to certain limitations. First, it exclusively analyzed data from the Web of Science Core Collection, potentially limiting the comprehensiveness of the final results. Future analyses should consider examining additional databases to enhance comprehensiveness. Second, despite efforts to include comprehensive search terms, it remains challenging to ascertain if all literature relevant to this topic was identified. Furthermore, the focus on English-language papers hinders our ability to assess research on physiotherapy applied to knee osteoarthritis in non-English-speaking countries. Lastly, it’s important to note that the number of citations and article centrality may fluctuate if conducted at different time points. Consequently, this study represents research conducted specifically from 2013 to 2022.

## 6 Conclusion

This study provides a comprehensive bibliometric analysis within this research field of publications, countries, journals, institutions, authors, references, and keywords to elucidate the current research status, trends, and hotspots. The analysis shows fluctuating research interest from 2013 to 2022, peaking in 2020. Developed countries lead this research, with Harvard University being the most influential institution. Enhanced collaboration among countries and institutions is necessary. High-impact journals prominently publish relevant papers, and notable collaborative networks exist among authors.

Research findings underscore that aerobic exercise and lower limb plyometrics are effective exercise therapies for KOA. Personalized dosage adjustments are recommended. Physical factor therapy can alleviate KOA symptoms, but high-quality clinical validation is necessary for efficacy assessment. Future prospects include remotely guided exercise and physical factor therapy via the Internet, promoting patient self-management and reducing the medical burden while effectively mitigating KOA symptoms. Strengthening interdisciplinary collaboration is crucial for advancing research and applications, providing better treatment options for KOA patients.
