# Supplementary material for: A bibliometric analysis of the application of physical therapy in knee osteoarthritis from 2013 to 2022
Source: Front Med (Lausanne). 2024 Sep 3;11:1418433. doi: 10.3389/fmed.2024.1418433 (PMC11405238; doi:10.3389/fmed.2024.1418433)
Supplement: Supplementary file 1 [file Data_Sheet_1.PDF]

Supplementary Material

1.Supplementary Tables

TABLE 1 The Topic Search Query

Set	Results	Search Query
#1	60,130	(TS=((Modalities, Physical Therapy) OR(Modality, Physical Therapy) OR (Physical Therapy Modality) OR(Physiotherapy (Techniques)) OR (Physiotherapies (Techniques)) OR(Physical Therapy Techniques) OR (Physical Therapy Technique) OR (Techniques, Physical Therapy) OR (Group Physiotherapy) OR (Group Physiotherapies) OR (Physiotherapies, Group) OR (Physiotherapy, Group) OR (Physical Therapy) OR (Physical Therapies) OR (Therapy, Physical) OR (Neurological Physiotherapy)OR(Physiotherapy, Neurological) OR(Neurophysiotherapy))) Indexes=Web of Science, Timespan=20132022
#2	36,104	(TS=((Osteoarthritis, Knee) OR (Knee Osteoarthritis) OR (Knees Osteoarthritis) OR (Osteoarthritis, Knees) OR (Osteoarthritis of knee) OR (Osteoarthritis Of Knees) OR (Knee, Osteoarthritis Of) OR (Knees, Osteoarthritis Of))) Indexes=Web of Science, Timespan=20132022
#3	1,852	#1AND#2

TABLE 2 Top 10 countries/regions and Institutions

Rank	Country	Year	Count (%)	Centrality	Institution	Year	Count (%)	Centrality
1	USA	2013	412（30.36%）	0.56	University of Melbourne（Australia）	2013	72（5.31%）	0.13
2	Australia	2013	151（11.13%）	0.16	University of Southern Denmark（Denmark）	2013	43（3.17%）	0.03
3	China	2013	120（8.84%）	0.08	Duke University（USA）	2013	41（3.02%）	0.06

Continued form TABLE 2

Rank	Country	Year	Count (%)	Centrality	Institution	Year	Count (%)	Centrality
4	England	2013	106（7.81%）	0.18	Harvard University（USA）	2013	38（2.80%）	0.14
5	Canada	2013	86（6.34%）	0.04	US Department of Veterans Affairs（USA）	2013	36（2.65%）	0.04
6	Netherlands	2013	76（5.60%）	0.13	Monash University（Australia）	2014	34（2.51%）	0.03
7	Brazil	2013	74（5.45%）	0	Veterans Health Administration（USA）	2013	33（2.43%）	0.02
8	Turkey	2013	70（5.16%）	0	University of North Carolina（USA）	2013	32（2.36%）	0.04
9	Denmark	2013	70（5.16%）	0.01	University of North Carolina Chapel Hill（USA）	2015	29（2.14%）	0.03
10	Germany	2013	60（4.42%）	0.06	Pennsylvania Commonwealth System of Higher Education（USA）	2013	27（1.99%）	0.02

TABLE 3 Top 10 journals and co-cited journals

Rank	Journal	Count(%)	IF (2022)	JCR	Co-cited journal	Citation	IF (2022)	JCR
1	BMC Musculoskeletal Disorders	89(6.49%)	2.3	Q2	Osteoarthritis and Cartilage	974	7.0	Q1
2	Arthritis Care & Research	40(2.92%)	4.7	Q2	Arthritis Care & Research	857	4.7	Q1
3	BMJ Open	34(2.48%)	2.9	Q2	Annals of the Rheumatic Diseases	768	27.4	Q1
4	Osteoarthritis and Cartilage	33(2.41%)	7.0	Q1	Journal of Rheumatology	675	3.9	Q2
5	Trials	33(2.41%)	2.5	Q3	BMC Musculoskeletal Disorders	647	2.3	Q2
6	Clinical Rehabilitation	32(2.33%)	3.0	Q1	Physical Therapy	568	3.2	Q1
7	Archives of Physical Medicine And Rehabilitation	30(2.19%)	4.3	Q1	Arthritis & Rheumatology	495	13.3	Q1
8	Physical Therapy	29(2.11%)	3.2	Q1	Archives of Physical Medicine and Rehabilitation	492	4.3	Q1

Continued form TABLE 3

Rank	Journal	Count(%)	IF (2022)	JCR	Co-cited journal	Citation	IF (2022)	JCR
9	Journal of Orthopaedic & Sports Physical Therapy	26(1.90%)	6.1	Q1	Journal of Orthopaedic & Sports Physical Therapy	459	6.1	Q1
10	Journal of Arthroplasty	25(1.82%)	3.5	Q1	Cochrane Database of Systematic Reviews	434	8.4	Q1

TABLE 4 Top 10 authors and co-cited authors

Rank	Author	Count (%)	Centrality	Co-cited author	Citation	Centrality
1	Bennell, Kim L	40（2.95%）	0.07	Bellamy N	374	0.01
2	Hinman, Rana S	34（2.51%）	0.02	Anonymous	287	0.01
3	Roos, Ewa M	22（1.62%）	0.05	Fransen M	274	0.04
4	Allen, Kelli D	19（1.40%）	0.01	Altman R	259	0.01
5	Kasza, Jessica	12（0.88%）	0	Bennell KL	240	0.03
6	Dekker, Joost	12（0.88%）	0.01	McAlindon TE	230	0.07
7	Harvey, William F	10（0.74%）	0.01	Kellgren JH	229	0
8	Abbott, J Haxby	10（0.74%）	0.02	Zhang W	212	0.03
9	Risberg, May Arna	10（0.74%）	0	Roos EM	199	0.04
10	Katz, Jeffrey N	10（0.74%）	0	Hochberg MC	195	0.04

TABLE 5 Top 10 co-cited references

Rank	Reference	Author	Journal	Year	Count	Centrality
1	OARSI guidelines for the non-surgical management of knee osteoarthritis	McAlindon TE	Osteoarthritis Cartilage	2014	106	0.06
2	OARSI guidelines for the non-surgical management of knee, hip, and polyarticular osteoarthritis	Bannuru RR	Osteoarthritis Cartilage	2019	88	0.05

Continued form TABLE 5

Rank	Reference	Author	Journal	Year	Count	Centrality
3	American College of Rheumatology 2012 recommendations for the use of nonpharmacologic and pharmacologic therapies in osteoarthritis of the hand, hip, and knee	Hochberg MC	Arthritis Care Res	2012	71	0.07
4	2019 American College of Rheumatology/Arthritis Foundation Guideline for the Management of Osteoarthritis of the Hand, Hip, and Knee	Kolasinski SL	Arthritis Rheumatol	2020	67	0
5	EULAR recommendations for the non-pharmacological core management of hip and knee osteoarthritis	Fernandes L	Ann Rheum Dis	2013	60	0.02
6	Exercise for osteoarthritis of the knee	Fransen M	Cochrane Db Syst Rev	2015	51	0.13
7	Exercise for osteoarthritis of the knee: a Cochrane systematic review	Fransen M	Br J Sports Med	2015	49	0.01
8	Good Life with osteoArthritis in Denmark (GLA:D™): evidence-based education and supervised neuromuscular exercise delivered by certified physiotherapists nationwide F10	Skou ST	Bmc Musculoskel Dis	2017	41	0.11
9	The global burden of hip and knee osteoarthritis: estimates from the global burden of disease 2010 study	Cross M	Ann Rheum Dis	2014	37	0.08
10	Osteoarthritis	Hunter DJ	Lancet	2019	35	0.04

TABLE 6 Top 20 keywords

Rank	Keywords	Count	Centrality	Rank	Keywords	Count	Centrality
1	knee osteoarthritis	434	0.2	11	arthritis	124	0.53
2	hip	354	0.15	12	Outcome	120	0.03
3	management	290	0.37	13	reliability	120	0.14
4	osteoarthritis	276	0.08	14	physical activity	115	0.21
5	pain	274	0.14	15	recommendations	114	0.51
6	physical therapy	236	0.06	16	exercise therapy	103	0.61
7	therapy	205	0.03	17	knee	102	0.06

Continued form TABLE 6

8	exercise	176	0.29	18	physical function	101	0.2
9	older adults	146	0.15	19	rehabilitation	99	0.37
10	quality of life	131	0.26	20	efficacy	93	0.03

2.Supplementary Figures

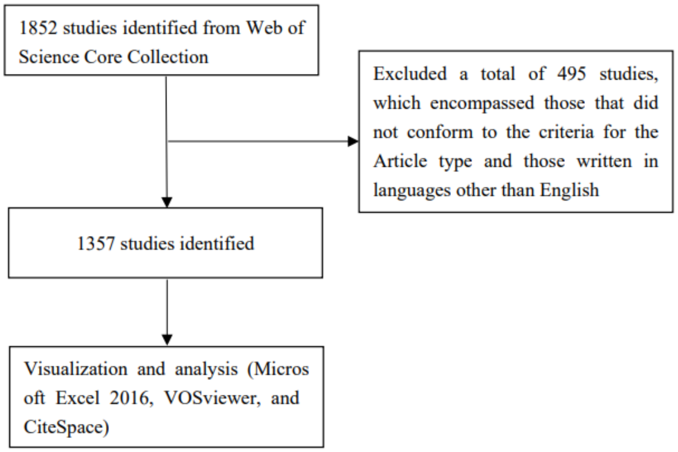

FIGURE 1 Flowchart of literature selection.

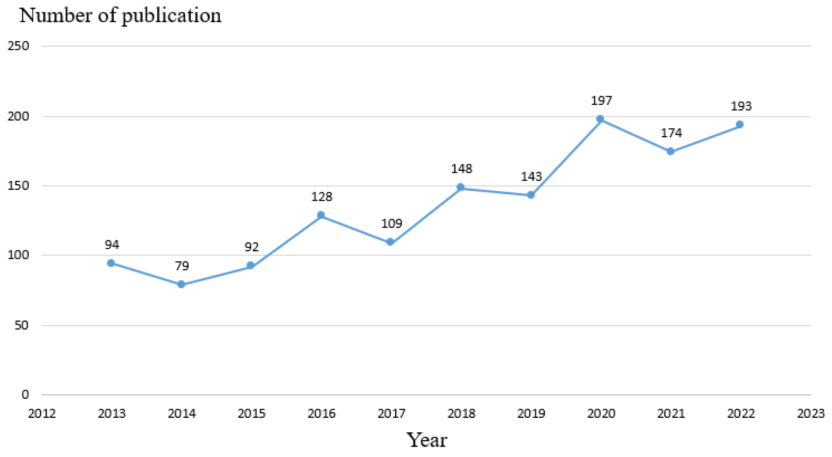

FIGURE 2 Trends in publications over the past 10 years.

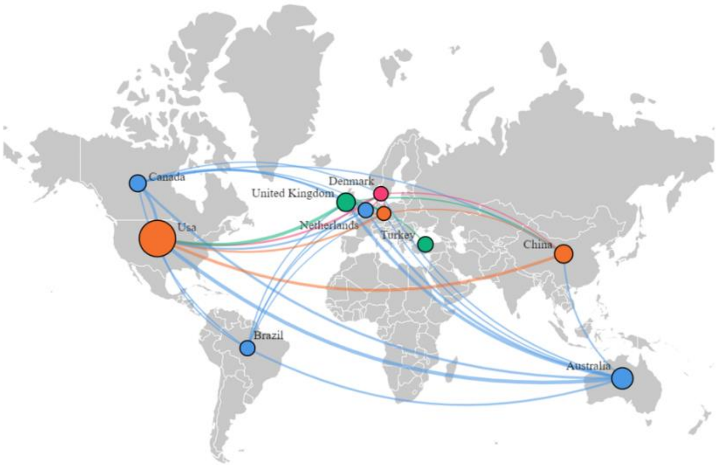

FIGURE 3 World map of top ten countries in terms of number of posts published.

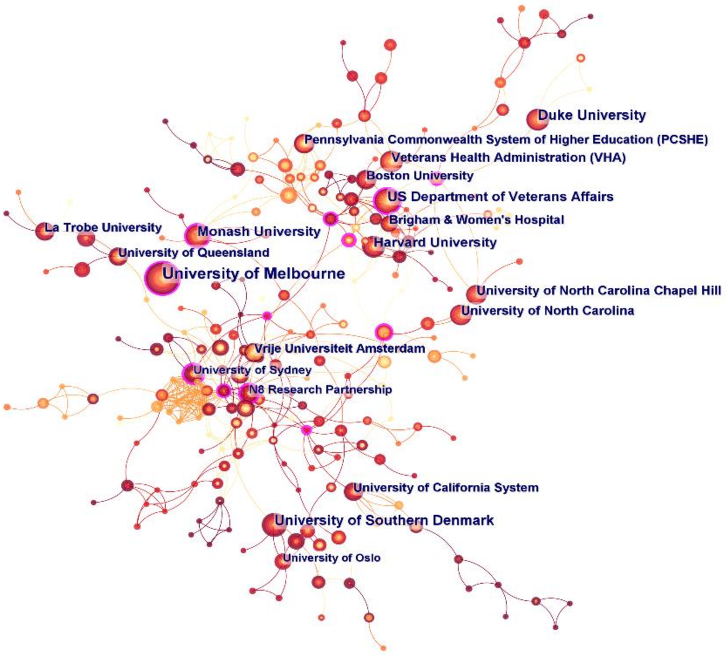

FIGURE 4 Distribution of publications from different institutions.

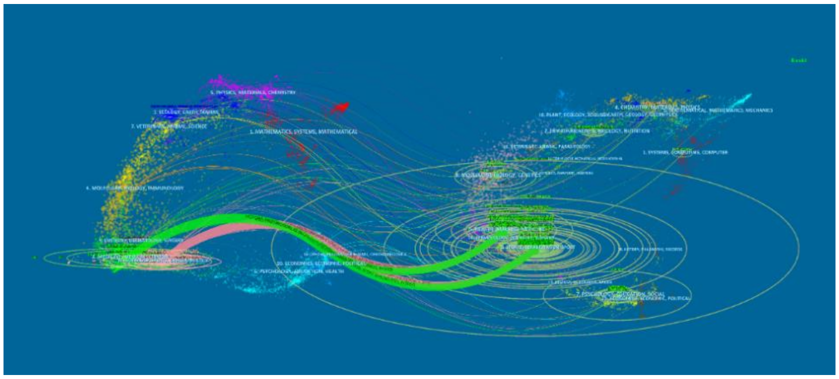

FIGURE 5 The dual-map overlay of journals.

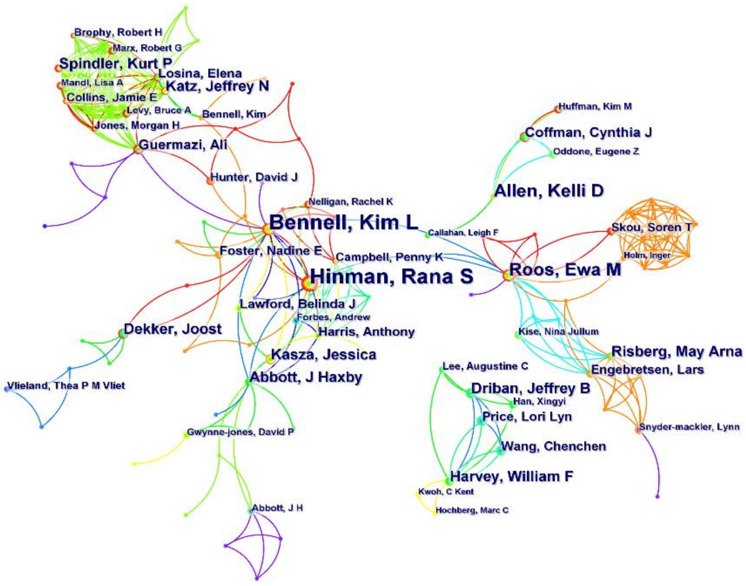

FIGURE 6 CiteSpace visualization map of authors.

Top 30 References with the Strongest Citation Bursts

References	Year	Strength	Begin	End	2013 - 2022
Zhang W, 2010, OSTEOARTHR CARTILAGE, V18, P476, DOI 10.1016/j.joca.2010.01.013, DOI	2010	14.74	**2013**	2015	
Zhang W, 2008, OSTEOARTHR CARTILAGE, V16, P137, DOI 10.1016/j.joca.2007.12.013, DOI	2008	11.19	**2013**	2013	
Fransen M, 2009, J RHEUMATOL, V36, P1109, DOI 10.3899/jrheum.090058, DOI	2009	5.84	**2013**	2014	
Bennell KL, 2011, J SCI MED SPORT, V14, P4, DOI 10.1016/j.jsams.2010.08.002, DOI	2011	5.39	**2013**	2016	
Hochberg MC, 2012, ARTHRIT CARE RES, V64, P465, DOI 10.1002/acr.21596, DOI	2012	19.34	**2014**	2017	
Fernandes L, 2013, ANN RHEUM DIS, V72, P1125, DOI 10.1136/annrheumdis-2012-202745, DOI	2013	14.96	**2014**	2018	
Katz JN, 2013, NEW ENGL J MED, V368, P1675, DOI 10.1056/NEJMoa1301408, DOI	2013	5.25	**2014**	2016	
Dobson F, 2013, OSTEOARTHR CARTILAGE, V21, P1042, DOI 10.1016/j.joca.2013.05.002, DOI	2013	7.66	**2015**	2018	
Cross M, 2014, ANN RHEUM DIS, V73, P1323, DOI 10.1136/annrheumdis-2013-204763, DOI	2014	9.97	**2016**	2019	
Bennell KL, 2014, BEST PRACT RES CL RH, V28, P93, DOI 10.1016/j.berh.2014.01.009, DOI	2014	6.29	**2016**	2018	
McAlindon TE, 2014, OSTEOARTHR CARTILAGE, V22, P363, DOI 10.1016/j.joca.2014.01.003, DOI	2014	25.56	**2017**	2019	
Fransen M, 2015, COCHRANE DB SYST REV, V0, P0, DOI 10.1002/14651858.CD004376.pub3, DOI	2015	10.6	**2017**	2020	
Nelson AE, 2014, SEMIN ARTHRITIS RHEU, V43, P701, DOI 10.1016/j.semarthrit.2013.11.012, DOI	2014	9.05	**2017**	2018	
Juhl C, 2014, ARTHRITIS RHEUMATOL, V66, P622, DOI 10.1002/art.38290, DOI	2014	8.89	**2017**	2019	
Messier SP, 2013, JAMA-J AM MED ASSOC, V310, P1263, DOI 10.1001/jama.2013.277669, DOI	2013	5.54	**2017**	2018	
Fransen M, 2015, BRIT J SPORT MED, V49, P0, DOI 10.1136/bjsports-2015-095424, DOI	2015	14.13	**2018**	2020	
McAlindon TE, 2015, OSTEOARTHR CARTILAGE, V23, P747, DOI 10.1016/j.joca.2015.03.005, DOI	2015	6.74	**2018**	2020	
Skou ST, 2015, NEW ENGL J MED, V373, P1597, DOI 10.1056/NEJMoa1505467, DOI	2015	6.08	**2018**	2020	
Abbott JH, 2013, OSTEOARTHR CARTILAGE, V21, P525, DOI 10.1016/j.joca.2012.12.014, DOI	2013	5.56	**2018**	2018	
Bennell KL, 2017, ANN INTERN MED, V166, P453, DOI 10.7326/M16-1714, DOI	2017	5.47	**2018**	2022	
Kise NJ, 2016, BMJ-BRIT MED J, V354, P0, DOI 10.1136/bmj.i3740, DOI	2016	6.83	**2019**	2022	
Skou ST, 2017, BMC MUSCULOSKEL DIS, V18, P0, DOI 10.1186/s12891-017-1439-y, DOI	2017	5.81	**2019**	2022	
Hunter DJ, 2019, LANCET, V393, P1745, DOI 10.1016/S0140-6736(19)30417-9, DOI	2019	11.9	**2020**	2022	
da Costa BR, 2017, LANCET, V390, PE21, DOI 10.1016/S0140-6736(17)31744-0, DOI	2017	5.63	**2020**	2022	
Bannuru RR, 2019, OSTEOARTHR CARTILAGE, V27, P1578, DOI 10.1016/j.joca.2019.06.011, DOI	2019	33.82	**2021**	2022	
Kolasinski SL, 2020, ARTHRITIS RHEUMATOL, V72, P220, DOI 10.1002/art.41142, DOI	2020	24.62	**2021**	2022	
Deyle GD, 2020, NEW ENGL J MED, V382, P1420, DOI 10.1056/NEJMoa1905877, DOI	2020	7.54	**2021**	2022	
Wallace IJ, 2017, P NATL ACAD SCI USA, V114, P9332, DOI 10.1073/pnas.1703856114, DOI	2017	6.59	**2021**	2022	
Hurley M, 2018, COCHRANE DB SYST REV, V0, P0, DOI 10.1002/14651858.CD010842.pub2, DOI	2018	6.59	**2021**	2022	
Bruyere O, 2019, SEMIN ARTHRITIS RHEU, V49, P337, DOI 10.1016/j.semarthrit.2019.04.008, DOI	2019	5.17	**2021**	2022	

FIGUER 7 CiteSpace visualization map of top 30 references with the strongest citation bursts.

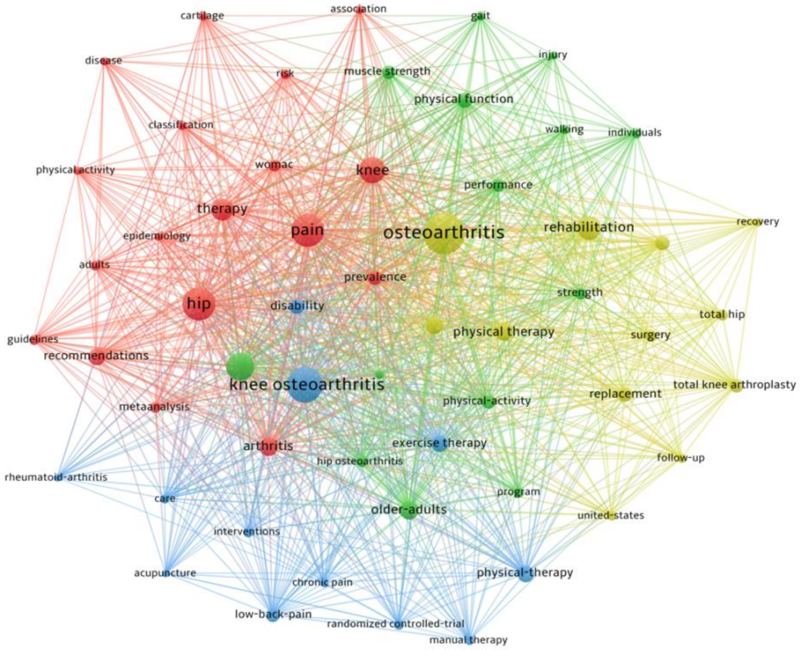

FIGURE 8 CiteSpace visualization map of keywords clustering analysis.

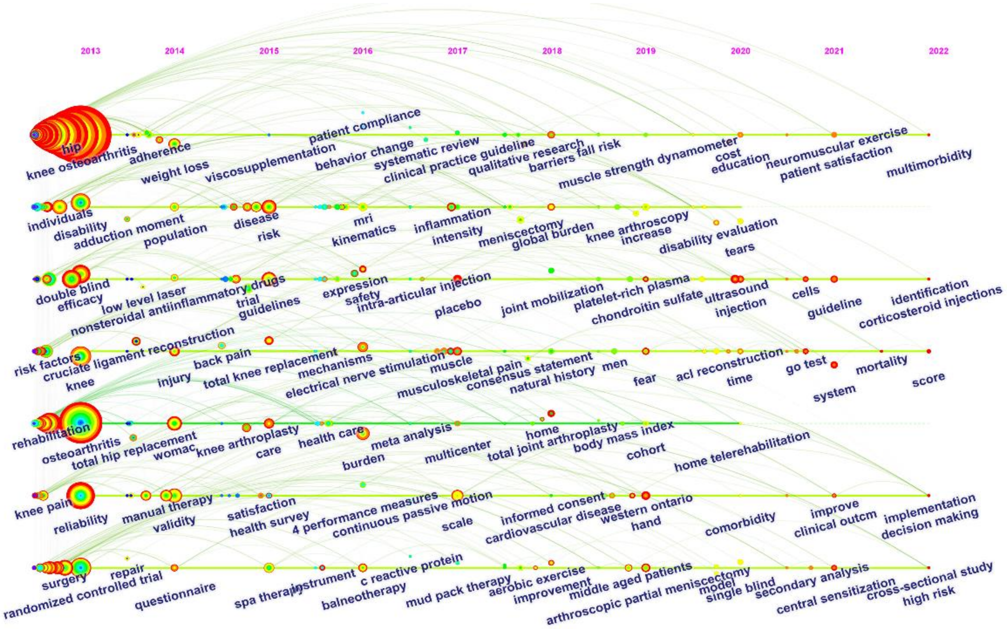

FIGURE 9 CiteSpace visualization map of timeline viewer related to Keyword.
